# Effects of Periarticular Multimodal Drug Injection on Pain Control, Early Mobilization, and Length of Hospital Stay in Patients Undergoing Total Knee Arthroplasty

**DOI:** 10.3390/life14081018

**Published:** 2024-08-15

**Authors:** Jian-Jiun Chen, Yun-Che Wu, Chuan-Yu Hung, Cheng-Hung Lee, Jun-Sing Wang

**Affiliations:** 1 Department of Orthopedics and Traumatology, Taipei Veterans General Hospital, Taipei 11217, Taiwan; jianjiunchen@gmail.com; 2 Department of Orthopedics, Taichung Veterans General Hospital, Taichung 407219, Taiwan; wcmin0806@gmail.com (Y.-C.W.); leechenghung0115@gmail.com (C.-H.L.); 3 Department of Pharmacy, Taichung Veterans General Hospital, Taichung 407219, Taiwan; chuanyu@vghtc.gov.tw; 4 Department of Food Science and Technology, Hung Kuang University, Taichung 433304, Taiwan; 5 Department of Post-Baccalaureate Medicine, College of Medicine, National Chung Hsing University, Taichung 402202, Taiwan; 6 Division of Endocrinology and Metabolism, Department of Internal Medicine, Taichung Veterans General Hospital, Taichung 407219, Taiwan

**Keywords:** hospital stay, orthopedics, pain control, periarticular injection, total knee arthroplasty

## Abstract

We investigated the effects of periarticular multimodal drug injection (PMDI) on postoperative pain control, patients’ mobilization, and length of hospital stay in patients undergoing total knee arthroplasty (TKA). We retrospectively enrolled patients who underwent unilateral TKA between 2019 and 2020. The formula for PMDI included 0.5 mL epinephrine (1 mg/mL), 1 mL ketorolac (30 mg/mL), 0.5 mL morphine (10 mg/mL), and 20 mL bupivacaine hydrochloride (5 mg/mL), mixed with 60 mL normal saline. The outcomes of interest included (1) the amount of patient-controlled anesthesia (PCA) consumption in the first 24 h after the surgery, (2) early mobilization within 24 h after the surgery, and (3) the length of hospital stay. A total of 127 patients were analyzed. Compared with patients who did not receive PMDI, those who received PMDI had lower consumption of PCA in the first 24 h (β coefficient −29.9, 95% CI −51.9 to −7.9, *p* = 0.008), higher odds of early mobilization within 24 h (odds ratio 8.263, 95% CI 3.041 to 22.453, *p* < 0.001), and shorter length of hospital stay (β coefficient −0.705, 95% CI −1.158 to −0.252, *p* = 0.003). We suggest that PMDI may be considered for patients undergoing TKA to improve the quality of care and shorten their length of hospital stay.

## 1. Introduction

Total knee arthroplasty (TKA) is one of the most common orthopedic procedures. In the United States, approximately 450,000 primary TKA surgeries were performed in 2005, with an estimated increase to 3.48 million by 2030 [1]. Despite its prevalence, postoperative pain management remains a significant challenge [2,3,4]. Effective pain control is crucial for facilitating functional recovery and improving the quality of care after surgery [5,6].

Various approaches to postoperative pain management have been explored [7], yet progress has been unsatisfactory. Current standard management includes the use of anesthetics and analgesics administered through oral, intravenous, epidural, and local methods [1]. For TKA patients, local infiltration analgesia, also known as periarticular multimodal drug injection (PMDI), has been proposed to enhance pain management and reduce side effects associated with systemic medication [8,9,10].

PMDI, which combines multiple medications for local infiltration, may provide more effective pain relief and allow for lower dosages of each drug [2]. However, the optimal regimen for PMDI is still uncertain. While most studies suggest that PMDI improves postoperative pain control in TKA patients [9,11,12,13,14], some reports show inconsistent results [15]. Despite its potential benefits, PMDI has not become a routine procedure in TKA due to several factors: the lack of large-scale, high-quality randomized controlled trials; concerns about potential side effects such as local tissue reactions and systemic toxicity; and variability in PMDI composition and administration across studies [12,16].

Given that PMDI has demonstrated benefits in reducing postoperative pain and enhancing functional recovery, it may also help shorten the length of hospital stay. However, few studies have specifically investigated this aspect, and their results have been inconsistent. Therefore, in this study, we investigated the effects of PMDI on postoperative pain control, early mobilization, and length of hospital stay in TKA patients.

## 2. Materials and Methods

We retrospectively enrolled patients with primary knee osteoarthritis who underwent unilateral TKA between 2019 and 2020. All patients in this study received cemented total knee arthroplasties. We excluded patients with an age ≥ 90 years or with a history of drug abuse or allergy to analgesics. Patients with a history of stroke or major neurological deficit were also excluded because they might have impaired muscle power or altered pain perception, which may confound our assessment of PMDI efficacy. Patients with psychological problems or cognitive impairment, which may confound the process of pain assessment, were also excluded. This study was conducted in accordance with the Declaration of Helsinki. The study protocol was approved (approval number: CE22258A) by the Institutional Review Board of Taichung Veterans General Hospital, Taichung, Taiwan. Informed consent was waived because this was a retrospective study, and anonymized data were used for analyses.

The surgical procedures were performed by 9 board-certified, experienced orthopedic surgeons following a standardized procedure to minimize variability. The duration of a TKA surgery typically takes around one hour at our hospital. The decision to conduct PMDI or not was at the discretion of the treating surgeon. Surgeons who were more experienced with PMDI and had observed its benefits in previous cases were more likely to utilize it. Conversely, some surgeons who were either skeptical of its efficacy or concerned about potential side effects may have chosen not to use it. The formula for PMDI included 0.5 mL epinephrine (1 mg/mL), 1 mL ketorolac (30 mg/mL), 0.5 mL morphine (10 mg/mL), and 20 mL bupivacaine hydrochloride (5 mg/mL), mixed with 60 mL normal saline [17,18,19]. PMDI was performed before capsular repair. About 15 mL of the formula was injected around the posterior capsule, medial collateral ligament, and pes anserinus tendon sheath, followed by 10 mL injected into the infrapatellar fat pad, suprapatellar synovium, and patellar tendon sheath. Last, the remaining formula was injected into the incision site. After the surgery, patients were free to adopt patient-controlled anesthesia (PCA). The formula for PCA was a mixture of ropivacaine (1.6 mg/mL) and fentanyl (1.5 µg/mL) in normal saline. Patients were allowed to become mobile as tolerated after the surgery.

Patients’ medical history and medical treatments (including pre- and post-operative use of analgesics at outpatient clinics and post-operative use of antiplatelet drugs and anticoagulants) were recorded with electronic health records. All patients were evaluated using the American Society of Anesthesiologists (ASA) Physical Status Classification System [20], and only those classified as ASA I or II were included in the analyses. Only patients with Kellgren–Lawrence grade [21] III–IV underwent surgery. Additionally, we recorded and compared baseline characteristics such as age, sex, and BMI between the groups. Patients’ perception of pain was assessed using Visual Analogue Scale (VAS) [21] before (within 24 h) and after (within 24 h) the surgery. The outcomes of interest in this study included (1) amount of PCA consumption in the first 24 h after the surgery, (2) early mobilization (could walk with a walker independently within 24 h) after the surgery, and (3) length of hospital stay. We examined whether PMDI was independently associated with these outcomes after adjustment for patients’ comorbidities. These outcome measures were chosen to assess the effectiveness of PMDI on postoperative pain control, early mobilization, and reduced hospital stay.

All statistical analyses were conducted using the Statistical Package for the Social Sciences (IBM SPSS version 22.0; International Business Machines Corp., New York, NY, USA). Between-group differences in continuous and categorical variables were examined using independent *t*-test and Chi-square test, respectively. The associations between PMDI and PCA consumption amount within 24 h and length of hospital stay were examined using linear regression analysis. PCA consumption within 24 h was measured in milliliters of morphine equivalent, and length of hospital stay was recorded from one day before the surgery to the day patients were discharged. Logistic regression analysis was used to determine the association between PMDI and the percentage of patients who were able to begin walking with or without assistance within 24 h after the surgery. Age, sex, body mass index, smoking, and patients’ comorbidities were adjusted in the linear and logistic regression models. Statistical significance was determined with a two-sided *p* value of less than 0.05.

## 3. Results

A total of 127 patients were analyzed. Table 1 shows the characteristics of the study population. There were no significant differences in clinical variables, the preoperative use of analgesics, operation time, and the postoperative use of anti-platelet/anti-coagulants and PCA between patients who received PMDI (*n* = 39) and those who did not (*n* = 88).

Patients’ outcomes of interest are shown in Table 2. Patients who received PMDI had a lower amount of PCA use in the first 24 h (126 ± 47 vs. 155 ± 44 mL, *p* = 0.009), a higher rate of early mobilization within 24 h (84.6% vs. 43.2%, *p* < 0.001), and a shorter length of hospital stay (5.4 ± 1.0 vs. 6.2 ± 1.3 days, *p* = 0.001), compared with those who did not. There were no significant between-group differences in terms of pain scores (VAS before and after the operation) and chronic use of analgesics (3 months after the surgery). Regarding side effects, a higher rate of patients who received PMDI reported skin itching/allergy (17.9% vs. 5.7%, *p* = 0.029).

We examined the associations between PMDI and patients’ outcomes. PMDI was associated with a lower consumption of PCA in the first 24 h after the surgery (β coefficient −28.7, 95% CI −49.9 to −7.4, *p* = 0.009, Table 3). The association remained significant after adjustment for age, sex, and patients’ comorbidities (β coefficient −29.9, 95% CI −51.9 to −7.9, *p* = 0.008, Table 3). Moreover, PMDI was independently associated with higher odds of early mobilization within 24 h after the surgery (odds ratio 8.263, 95% CI 3.041 to 22.453, *p* < 0.001, Table 4). Finally, PMDI was independently associated with a shorter length of hospital stay (β coefficient −0.705, 95% CI −1.158 to −0.252, *p* = 0.003, Table 5).

## 4. Discussion

In this retrospective study, we demonstrated that PMDI in patients who underwent unilateral TKA was independently associated with lower consumption of PCA, higher odds of early mobilization within 24 h after the surgery, and a shorter length of hospital stay. This is in view of the fact that PMDI was associated with only a modest increase in the rate of skin itching/allergy, which refers to localized allergic reactions at the injection site, such as redness, itching, or swelling. These reactions were mild and resolved after treatment with antihistamines or corticosteroids within 3 days. There were no reports of serious allergic reactions or systemic toxicity. Our findings suggest that PMDI might be considered for patients undergoing TKA to improve the quality of care and shorten the length of hospital stay. However, we acknowledged that the possibility of toxicity exists, particularly if the patients have pre-existing allergies to any of the components.

Appropriate pain control after surgical procedures may improve the quality of care and probably shorten the length of hospital stay [5,6]. PMDI likely exerts its effects through a combination of local anesthetic action, anti-inflammatory properties, and analgesic effects of its components. Bupivacaine provides prolonged local anesthesia, ketorolac reduces inflammation, morphine offers opioid analgesia, and epinephrine prolongs the effects of these drugs by reducing systemic absorption. This multimodal approach may synergistically contribute to the benefit of pain relief, early mobilization, and shorter length of hospital stay [12,22]. Similar to previous studies [23,24], we observed that PMDI was associated with lower consumption of PCA after surgery (β coefficient −29.9, 95% CI −51.9 to −7.9, *p* = 0.008, Table 3). The higher consumption of PCA in patients with no PMDI may help explain why no significant between-group difference in postoperative pain scores was found in our study. Moreover, we found that PMDI was associated with higher odds of early mobilization within 24 h after the surgery (odds ratio 8.263, 95% CI 3.041 to 22.453, *p* < 0.001, Table 4) and a shorter length of hospital stay (β coefficient −0.705, 95% CI −1.158 to −0.252, *p* = 0.003, Table 5). PMDI has been shown to improve quadricep function recovery in patients undergoing TKA [25,26]. This may facilitate early postoperative mobilization and discharge from the hospital.

Despite the benefit of PMDI for postoperative care, it has not yet become a routine procedure in patients undergoing TKA. The limited use of PMDI can be attributed to several factors, including the variations in the formula used for PMDI [19,27], and the lack of a large-scale randomized trial to prove the effects of PMDI, not only on pain control but also on other indices of healthcare quality. There are concerns about potential side effects and variability in the technique’s application. Although our study did not observe any serious adverse effects, several complications had been reported in the previous literature, such as prolonged numbness, local tissue irritation, and rare instances of systemic toxicity [28]. These potential risks may contribute to some surgeons’ reluctance to adopt PMDI. Our findings support the use of PMDI in patients undergoing TKA as it appears to increase the likelihood of early postoperative mobilization and discharge from the hospital. Furthermore, the safety profiles of PMDI are acceptable. There were no significant side effects of PMDI in most previous studies [10]. We found a modest increase in skin itching/allergy among patients who received PMDI, and the symptoms were self-limited. Hence, PMDI is well-tolerated according to our findings and previous studies.

The use of PMDI in TKA patients has significant clinical implications. It can lead to improvement of pain control, facilitating early mobilization and reducing the length of hospital stay. These outcomes can enhance patient recovery, reduce healthcare costs, and improve overall patient satisfaction. Our findings support the integration of PMDI into standard TKA protocols, with appropriate management of potential side effects. The higher rate of skin itching/allergy in the PMDI group is statistically significant and indicates a need for vigilance in managing these side effects. This balance is crucial for maximizing patient comfort and satisfaction. Future research could explore the optimization of PMDI formulations, the long-term outcomes of patients receiving PMDI, and its application in other types of orthopedic surgeries. Investigating the genetic and molecular mechanisms underlying individual responses to PMDI could also offer personalized pain management strategies. On the other hand, combining different pain management techniques might have better outcomes. For example, combining adductor canal block with infiltration between the popliteal artery and capsule of the knee and PMDI may yield superior results. Significant findings have been reported in some randomized controlled trials and meta-analyses [10,29,30]. Future research may explore different combinations to achieve optimal pain relief.

There were some limitations in this study. First, this was a retrospective study conducted in a single institution. Furthermore, the number of patients was relatively small. Second, the decision to perform PMDI or not was at the discretion of the treating surgeon. Some surgeons never used PMDI, and this factor might have confounded our results. Nevertheless, there were no significant differences in the baseline characteristics between the two groups (Table 1), and we conducted multivariate-adjusted analyses to investigate the effects of PMDI on the study outcomes (Table 3, Table 4 and Table 5). Third, the regimen of PMDI used in this study is not the same as in some previous studies [22]. This should be taken into account when interpreting our results. Despite these limitations, our observations support the benefit and safety of PMDI in postoperative care for patients undergoing TKA independent of patients’ comorbidities. A large-scale prospective study is needed to confirm our findings.

## 5. Conclusions

PMDI in patients who underwent unilateral TKA was associated with higher odds of early mobilization within 24 h after the surgery and a shorter length of hospital stay. Our findings suggest that PMDI may be considered for patients undergoing TKA to improve the quality of care and shorten the length of hospital stay.

## Figures and Tables

**Table 1:** Characteristics of the study population.

Variables	Periarticular Multimodal Drug Injection		
	No	Yes	*p* Value
N	88	39	
Age (years)	71.8 ± 9.0	71.7 ± 6.4	0.949
Male sex	22 (25.0)	12 (30.8)	0.498
Body mass index (kg/m^2^)	28.5 ± 4.5	28.0 ± 3.9	0.596
Smoking	3 (3.4)	2 (5.1)	0.646
Diabetes	27 (30.7)	13 (33.3)	0.767
Hypertension	59 (67.0)	25 (64.1)	0.746
Chronic kidney disease	24 (27.3)	8 (20.5)	0.418
Preoperative analgesics ^†^	68 (77.3)	30 (76.9)	0.965
Operation time (minutes)	135 ± 45	140 ± 34	0.600
Postoperative treatment			
Anti-platelet ^‡^	72 (81.8)	35 (89.7)	0.258
Anti-coagulants ^§^	15 (17.0)	4 (10.3)	0.322
Patient-controlled analgesia	79 (89.8)	34 (87.2)	0.667

Values are mean ± SD or *n* (%). ^†^ Non-steroidal anti-inflammatory drugs, opioids, or acetaminophen. ^‡^ Aspirin 100 mg daily. ^§^ Dabigatran 150 mg daily, rivaroxaban 10 mg daily, or edoxaban 30 mg daily.

**Table 2:** Patients’ outcomes according to periarticular multimodal drug injection.

Variables	PMDI		
	No	Yes	*p* Value
PCA consumption in the first 24 h (mL)	155 ± 44	126 ± 47	0.009
Early mobilization within 24 h	38 (43.2)	33 (84.6)	<0.001
Length of hospital stay (days)	6.2 ± 1.3	5.4 ± 1.0	0.001
Pain scores			
VAS before operation	1.7 ± 0.9	1.6 ± 0.7	0.523
VAS after operation	3.9 ± 2.4	3.6 ± 2.2	0.451
VAS postoperative day 1	3.9 ± 2.0	3.5 ± 1.9	0.414
VAS postoperative day 3	3.9 ± 2.6	3.7 ± 2.2	0.677
Side effects			
Constipation	35 (39.8)	14 (35.9)	0.679
Nausea/Vomiting	28 (31.8)	10 (25.6)	0.483
Urine retention	6 (6.8)	7 (17.9)	0.056
Skin itching/Allergy	5 (5.7)	7 (17.9)	0.029
Dizziness	7 (8.0)	5 (12.8)	0.387
Delirium	1 (1.1)	1 (2.6)	0.551
Use of analgesics 3 months after operation ^†^	43 (48.9)	17 (43.6)	0.583

Values are mean ± SD or *n* (%). PCA, patient-controlled analgesia. PMDI, periarticular multimodal drug injection. VAS, visual analog scale. ^†^ Non-steroidal anti-inflammatory drugs, opioids, or acetaminophen.

**Table 3:** Association of PMDI with PCA consumption (mL) in the first 24 h.

	β Coefficient (95% CI)	*p*
Independent variable: PMDI		
Model 1	−28.7 (−49.9, −7.4)	0.009
Model 2	−29.9 (−51.1, −8.6)	0.006
Model 3	−29.9 (−51.9, −7.9)	0.008

Model 1, unadjusted. Model 2, adjusted for age and sex. Model 3, adjusted for variables in Model 2 plus body mass index, smoking, diabetes, hypertension, and chronic kidney disease. PCA, patient-controlled analgesia. PMDI, periarticular multimodal drug injection.

**Table 4:** Association of PMDI with early mobilization within 24 h after the surgery.

	Odds Ratio (95% CI)	*p*
Independent variable: PMDI		
Model 1	7.237 (2.752, 19.028)	<0.001
Model 2	7.312 (2.772, 19.283)	<0.001
Model 3	8.263 (3.041, 22.453)	<0.001

Model 1, unadjusted. Model 2, adjusted for age and sex. Model 3, adjusted for variables in Model 2 plus body mass index, smoking, diabetes, hypertension, chronic kidney disease, and use of patient-controlled analgesia. PMDI, periarticular multimodal drug injection.

**Table 5:** Association of PMDI with length of hospital stay.

	β Coefficient (95% CI)	*p*
Independent variable: PMDI		
Model 1	−0.746 (−1.196, −0.296)	0.001
Model 2	−0.757 (−1.210, −0.304)	0.001
Model 3	−0.705 (−1.158, −0.252)	0.003

Model 1, unadjusted. Model 2, adjusted for age and sex. Model 3, adjusted for variables in Model 2 plus body mass index, smoking, diabetes, hypertension, chronic kidney disease, and use of patient-controlled analgesia. PMDI, periarticular multimodal drug injection.

## Data Availability

The datasets presented in this article are not readily available because of privacy restrictions. Requests to access the datasets should be directed to the corresponding author.
